# Chronic Kidney Disease Awareness Campaign and Mobile Health Education to Improve Knowledge, Quality of Life, and Motivation for a Healthy Lifestyle Among Patients With Chronic Kidney Disease in Bangladesh: Randomized Controlled Trial

**DOI:** 10.2196/37314

**Published:** 2022-08-11

**Authors:** Mohammad Habibur Rahman Sarker, Michiko Moriyama, Harun Ur Rashid, Md Moshiur Rahman, Mohammod Jobayer Chisti, Sumon Kumar Das, Samir Kumar Saha, Shams El Arifeen, Tahmeed Ahmed, A S G Faruque

**Affiliations:** 1 Nutrition and Clinical Services Division International Centre for Diarrhoeal Disease Research, Bangladesh Dhaka Bangladesh; 2 Graduate School of Biomedical and Health Sciences Hiroshima University Hiroshima Japan; 3 Kidney Foundation Hospital and Research Institute Dhaka Bangladesh; 4 Menzies - School of Health Research Charles Darwin University Darwin Australia; 5 Child Health Research Foundation Dhaka Bangladesh; 6 Dhaka Shishu Hospital Dhaka Bangladesh; 7 Maternal and Child Health Division International Centre for Diarrhoeal Disease Research, Bangladesh Dhaka Bangladesh

**Keywords:** Bangladesh, health education, health knowledge, quality of life, motivation, randomized controlled trial, RCT, campaign, chronic kidney disease, knowledge, mobile health, mHealth, kidney, chronic disease, chronic condition, patient education, patient knowledge, low- and middle-income countries, LMIC

## Abstract

**Background:**

Chronic kidney disease (CKD) is linked to major health consequences and a poor quality of life. Despite the fact that CKD is becoming more prevalent, public knowledge of the disease remains low.

**Objective:**

This study aimed to evaluate the outcome of a health education intervention designed to enhance knowledge, health-related quality of life (QOL), and motivation about healthy lifestyle among adults with CKD.

**Methods:**

This study was a parallel-group (1:1), randomized controlled trial in the Mirzapur subdistrict of Bangladesh that compared 2 groups of patients with CKD. Adults with CKD (stages 1-3) were enrolled in November 2020 and randomly assigned the intervention or control group. The intervention group received health education through a CKD awareness campaign and mobile health technologies and was observed for 6 months, whereas the control group received standard treatment. The primary outcome was the evaluation of improved scores on the CKD knowledge questionnaire, and the secondary outcomes were improved QOL and changes in the levels of blood pressure (BP), BMI, serum creatinine, fasting blood sugar (FBS), hemoglobin, cholesterol, high-density lipoprotein cholesterol, triglyceride, serum uric acid, blood urea nitrogen (BUN), and albumin-to-creatinine ratio.

**Results:**

The study enrolled 126 patients (control: n=63; intervention: n=63) and performed intention-to-treat analysis. The analyses included repeated measures ANOVA, and the results were observed to be significantly different from within groups (*P*<.001), between groups (*P*<.001), and the interaction of group × time factor (*P*<.001) for knowledge score. Diastolic BP and BMI showed significant differences arising from within groups (*P*<.001 and *P=*.01, respectively) and the interaction of group × time factor (*P*=.001 and *P=*.02, respectively); food salinity and hip circumferences showed significant differences arising from within groups (*P*=.001 and *P=*.03, respectively) and between groups (*P*=.001 and *P=*.02, respectively). Moreover, systolic BP and waist circumference showed significant differences from within groups (*P*<.001 and *P=*.003, respectively). However, no significant differences were found arising from within groups, between groups, and the interactions of group × time for QOL, urine salinity, and mid-upper arm circumference. Regarding the laboratory findings, from baseline to 6 months, the mean (SD) FBS decreased by 0.51 (3.77) mmol/L in the intervention group and 0.10 (1.44) mmol/L in the control group (*P*=.03); however, blood urea nitrogen increased by 3.64 (7.17) mg/dL in the intervention group and 1.68 (10.10) mg/dL in the control group (*P*=.01).

**Conclusions:**

The health education strategy, which included a campaign and mobile health, showed promise for enhancing CKD knowledge among patients with CKD. This strategy may also aid patients with CKD in controlling their FBS and BP. The combined health education initiatives give evidence for scaling them up in Bangladesh and possibly other low- and middle-income countries, particularly in rural and peri-urban settings.

**Trial Registration:**

ClinicalTrials.gov NCT04094831; https://clinicaltrials.gov/ct2/show/NCT04094831.

**International Registered Report Identifier (IRRID):**

RR2-10.2196/30191

## Introduction

Chronic kidney disease (CKD) is responsible for poor health outcomes, low quality of life (QOL), and high health care expenses [1]. Globally, the rising trend of CKD is being recognized as a future public health threat [2]. The prevalence of CKD in stages 1-3 has been documented at 8.9% of the global population [3], with rates higher in low-income nations such as India (15.6%) [4] and Bangladesh (21.33%) [5]. Early stage CKD is generally asymptomatic, and diagnosis is usually made through serum creatinine and albumin-to-creatinine ratio tests [6]. If left undetected and untreated, CKD can proceed to end-stage renal disease, which requires expensive renal replacement therapy such as dialysis or kidney transplantation to save the patient’s life [7]. Over the last decade, the economic burden of renal replacement therapy has increased dramatically and is substantially higher in low- and middle-income countries (LMIC) than in high-income countries [8]. Although primary renal disease causes CKD, the great majority of patients with CKD have concomitant health conditions such as diabetes, hypertension, and older age [9]. The majority of individuals with nonprimary renal disease are treated for associated risk factors such as diabetes and hypertension rather than the CKD itself [10]. Glomerular filtration rate declines slowly in most patients with CKD in stages 1-3; however, the declining trend varies among individuals and is influenced by a variety of factors such as diabetes, high blood pressure (BP), and older age, etc [6]. Individuals with CKD who receive proper information and knowledge about CKD and its risk factors are more likely to engage in health-promoting behaviors and lifestyle changes [11]. CKD early diagnosis and prevention strategies, such as a CKD preventive campaign, are currently being applied in a number of high-income countries. Increasing knowledge about CKD and its risk factors is a crucial strategy for slowing the disease’ progression.

In Bangladesh, community health workers (CHWs) are health cadres who work in public-sector health facilities. In recent years, CHWs in some places have begun to provide services for noncommunicable diseases, including health education and counseling [12]. The use of CHWs to deliver an education and monitoring intervention has been found to be effective in noncommunicable diseases such as reducing BP and has the potential to be scaled up in resource-limited settings [13,14]. They can make a substantial difference in the health of patients living with CKD. CHWs can educate patients to help protect their own kidneys and improve their QOL [15]. Mobile health (mHealth) is still in its implementation phase in the field of nephrology. However, mobile phone call–based health education has great potential to provide CKD knowledge and improve QOL, because it relies on basic mobile technology and requires limited literacy or numeracy skills [15]. A nephrologist-facilitated CKD health campaign also has the potential to improve patients’ knowledge and awareness [16,17].

The education of patients with CKD may increase perceived kidney disease knowledge among patients, improve QOL, and delay the progression of kidney disease [18,19]. A community-based screening revealed a high prevalence of CKD in stages 1-3, with only around 7% of the people being aware of their condition prior to the study; however, no health education intervention for these stages was investigated [5]. Most studies on the education of patients with CKD have focused on individuals with end-stage disease and shown improved outcomes [11]. However, a CKD campaign and mHealth-based health education in the early stages could be an integral part of patient management and the reduction of the related risk factors, slowing down the progression of the disease, and the need for such education is greater in rural and peri-urban areas. Thus, this study aimed to evaluate the outcome of a health education intervention designed to enhance knowledge, health-related QOL, and motivation about healthy lifestyle among rural and peri-urban adults with CKD (stages 1-3).

## Methods

### Design

**Figure 1:**
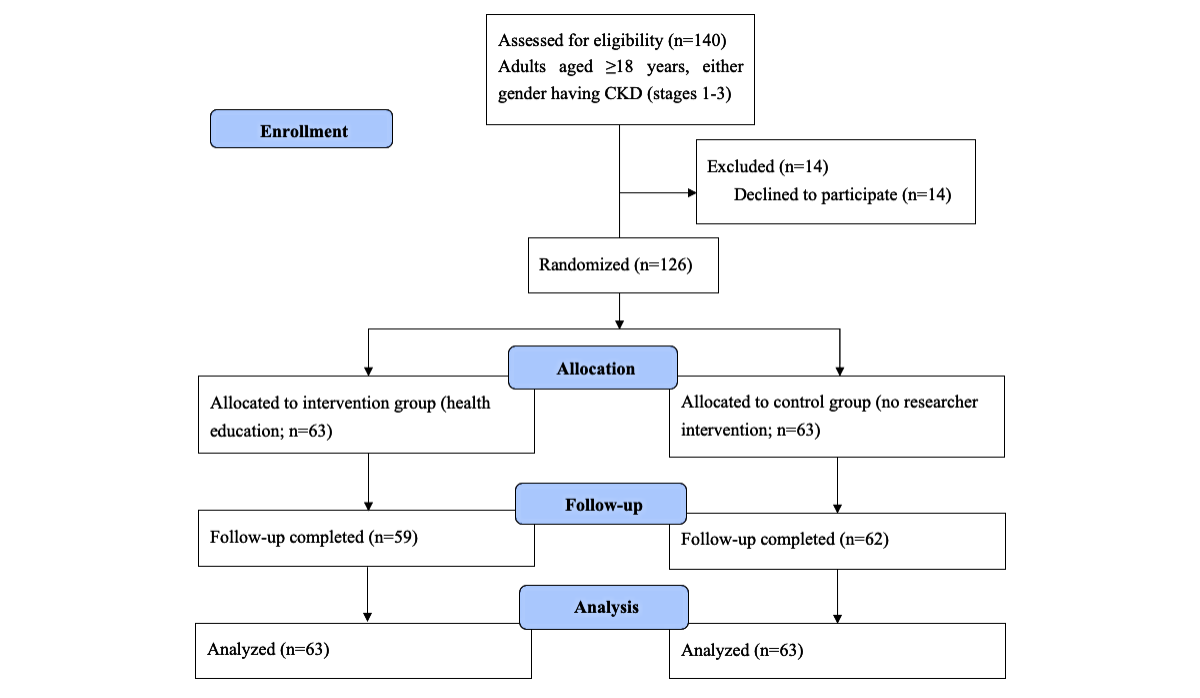
Flowchart of the participants throughout the study. CKD: chronic kidney disease.

This study was a community-based, single-center, prospective, open-label, parallel-group (1:1), randomized control trial (RCT) involving patients with CKD, conducted in a rural and peri-urban population of Bangladesh. This study was designed in accordance with the CONSORT (Consolidated Standards of Reporting Trials) [20] and SPIRIT (Standard Protocol Items: Recommendations for Interventional Trials) [21] guidelines. The study flowchart is shown in Figure 1. The total study duration was 1 year; during that 1 year, the intervention duration was 6 months, starting from mid-November 2020 and completed in May 2021.

### Study Population and Sampling

This study was conducted in the demographic surveillance system (DSS) area of the Mirzapur subdistrict under the Tangail district, which is 60 km north of Dhaka, the capital city of Bangladesh. The details of the Mirzapur DSS have been described in a previous publication [5]. The research team completed a CKD prevalence study before implementing this RCT [5]. The Chronic Kidney Disease Epidemiology Collaboration equation was used to estimate glomerular filtration rate [22], and the National Kidney Foundation Kidney Disease Outcomes Quality Initiative guidelines were used to define and indicate the stages of CKD [23]. We randomly enrolled study patients from the residents of the DSS who have been diagnosed with CKD. The inclusion and exclusion criteria are shown in Textbox 1.

**Inclusion and exclusion criteria.**

**Inclusion Criteria**

- Adults (aged ≥18 years) of either gender, diagnosed with chronic kidney disease in stages 1-3 (any one of the following: estimated glomerular filtration rate = 30-59 mL/min/1.73 m^2^ and albumin-to-creatinine ratio ≥30 mg/g for more than 3 months) [24]
- Lived continuously in the locality for at least 5 years
- Has a personal cell phone or access to a shared phone
- Gave written informed consent to participate in the study

**Exclusion Criteria**

- Individuals diagnosed with chronic kidney disease in stages 4 and 5
- Hospitalized at the time of enrollment
- Having any known, serious illness with questionable prognosis; for example, malignancy, mental illness, congenital disease, or gross physical disability (if they have prescriptions)
- Declined to give consent to participate in the study

### Randomization

Permuted block randomization technique was performed using a block size of 6 based on a computer-generated random number sequence. An experienced statistician, who is not involved in the study in any way, prepared the randomization table and listed the study patients’ numbers with the corresponding intervention allocations for patients with CKD in sequentially numbered, sealed envelopes according to the randomization schedule to correspond to the serial numbers of the patients with CKD. These envelopes were kept in an office locker. Allocation was concealed in identical sealed envelopes that were only opened when the study patient was ready for enrollment under the supervision of the principal investigator. This step took place after a patient with CKD had been enrolled in the study, after obtaining voluntary informed written consent and having been assigned a study number.

### Study Activities and Contents

CHWs conducted baseline home visits during the first week and received written informed consent before interviewing research patients and administering field-tested standardized questionnaires to collect information on socioeconomic and demographic characteristics, level of knowledge, and QOL. Next, they advised the study patients to visit Kumudini Women’s Medical College and Hospital’s laboratory for study-related investigations. After evaluating the study patients’ eligibility, the statistician performed the randomization. The responsible research staff then opened the sealed envelope, revealing the patient’s random allocation, and handed the patient’s ID number to the CHWs. Staff in this trial were not blinded to the intervention or control groups; however, they were blinded to the baseline interview. The CHWs called the enrolled trial patients assigned to the intervention group and invited them to the CKD awareness campaign.

In addition to baseline, at 3 and 6 months, the CHWs visited the patients’ homes for an interview and physical examination using the same questionnaire. After 6 months, the CHWs advised the study patients to visit the laboratory for study-related investigations.

During the home visits, the CHWs used a Portable Health Clinic box with the essential diagnostic equipment for this research (the details were described in our previous paper) [25]. At the baseline, they collected sociodemographic information such as age, gender, marital status, religion, occupation, educational background, income per month, patient’s current medical history including medication use, past medical history, and family history (3 generations) including current and immediate past medical history. The same information was collected at 3 and 6 months in case of any changes from the baseline. They also administered a questionnaire on CKD knowledge and QOL. Physical examinations were performed to measure BP, pulse, height, weight, waist and hip circumferences, and mid-upper arm circumference (MUAC). In addition, blood and spot urine samples were taken to estimate the kidney function status and their related risk factors such as serum creatinine, fasting blood sugar (FBS), hemoglobin, cholesterol, high-density lipoprotein cholesterol, triglyceride, serum uric acid, blood urea nitrogen (BUN), and albumin-to-creatinine ratio (Multimedia Appendix 1).

### Intervention Group

A CKD awareness campaign and mHealth technologies were used to provide health education to the intervention group.

#### CKD Awareness Campaign

A nephrologist conducted the CKD awareness campaign in the native language (Bengali). Important information related to CKD, such as the basics of kidney function and kidney diseases, stages, risk factors, and preventive measures were discussed during the half day campaign. Health education materials (leaflet, short textbook, and recording notebook) were provided to the study patients. The details of the CKD campaign have been discussed in the protocol paper [25].

#### mHealth Technology

A nephrologist trained the CHWs to conduct the mHealth education. The CHWs were trained over 3 days. The training was facilitated by a nephrologist and coordinated by the project’s principal investigator. The program included lectures, discussions, question-and-answer sessions, and role-playing. We developed training materials that were tied to the study objectives to ensure that the CHWs acquired the skills needed to deliver the education to the target patients. The CHWs conducted the health education over a mobile phone call using mHealth technology once every 2 weeks during the intervention period. They discussed with the study participants in the intervention group about fundamental kidney disease, risk factors, and CKD prevention methods. Over a 10-minute session, the patients were free to discuss their health-related concerns with the CHWs. The details of the contents have been described in the protocol paper [25] (Multimedia Appendix 2).

#### BP Check

During the intervention phase, CHWs performed weekly home visits to the patients belonging to the intervention group and measured their BP.

### Control Group

The control group received usual care and was observed without intervention throughout the trial period. At 3 and 6 months, an interview and physical examination were conducted for these patients. Furthermore, they returned to the laboratory for study-related investigations after 6 months.

### Sample Size

The sample size was calculated based on the primary outcome—the enhanced knowledge of the study patients following the intervention of the health education program. We assumed that the proportion of existing knowledge among patients with CKD (stages 1-3) was 40% [26], with the percentage of predicted knowledge increasing to 70% following the intervention. As a result, with 90% power and 20% loss to follow-up, the total sample size was 126 (63 in each group). The details of the sample size calculation have been described in the protocol paper [25].

### Study Outcomes

The primary and secondary outcomes were measured at baseline, 3 months, and 6 months for both the intervention and control groups (Textbox 2). The laboratory parameters were measured at baseline and 6 months.

**Primary and secondary outcomes.**

**Primary Outcome**

- Evaluation of improved scores on the Chronic Kidney Disease Knowledge Questionnaire [26]

**Secondary Outcomes**

- Improved quality of life; measured by the EQ-5D-5L quality of life questionnaire [27]
- Changes in the levels of blood pressure, BMI, serum creatinine, fasting blood sugar, hemoglobin, cholesterol, high-density lipoprotein cholesterol, triglyceride, serum uric acid, blood urea nitrogen, and albumin-to-creatinine ratio

### Measurements of Knowledge and QOL

The evaluation of knowledge was measured using the Kidney Knowledge questionnaire, a 24-item scale designed to assess the CKD knowledge of patients in stages 1-3. A more in-depth description of the method has been mentioned elsewhere [25]. QOL was measured using the standardized EQ-5D-5L questionnaire [28]. In this study, we used the Japanese region’s tariff to define the standard value (tariff) for EQ-5D-5L to assess the impact of the health care interventions on QOL.

### Statistical Analysis

In this study, intention-to-treat analysis was used to compare the outcomes of the intervention and control groups. Missing data were dealt with by using the last observation carried forward method [29]. All baseline indicators at the time of registration were analyzed to ensure the comparability of the randomized samples. For the baseline assessment, continuous variables were compared by 2-tailed *t* test or Mann-Whitney *U* test, and categorical variables were compared by chi-square test. Multiple comparisons were performed by 2-way repeated measures ANOVA for the evaluation of the outcome variables such as CKD knowledge questionnaire, physical measurements, and QOL at baseline, 3 months, and 6 months. In addition, generalized estimating equations were used to estimate the effect of the health education after adjusting for relevant covariates. However, outcome variables for laboratory findings were measured at baseline and 6 months. Changes in the laboratory profiles were compared between the intervention group and control group by 2-tailed *t* test or Mann-Whitney *U* test after checking the data normality. The significance level was set at *P*<.05. The statistical analyses were conducted using SPSS statistical software (version 20.0; IBM Corp).

### Consent to Participate

All study patients provided written informed consent and participated entirely voluntarily. The patients received a copy of the consent form. Each study patient received written information about the aim of the study and was informed that they could decide to leave the study at any time and for any reason. Patients in the intervention group were informed that they would get additional health education alongside the usual care, whereas patients in the control group were informed that they would not receive any interventions. Patients were assured that their information would not be disclosed, and they were informed about the use of data for analysis and the use of the results for enhancing patient care activities, conducting research, and publication without disclosing their name or identity.

### Ethics Approval

Ethical approval was obtained by the Research Review Committee and Ethical Review Committee of the International Centre for Diarrhoeal Disease Research, Bangladesh (icddr,b) (PR 19081). The study was registered at ClinicalTrials.gov (NCT04094831).

## Results

A total of 126 patients (control group: n=63; intervention group: n=63) were enrolled in the study. Of the 126 patients, 5 withdrew from further participation during the follow-up period—4 from the intervention group and 1 from the control group. Of the 4 patients who withdrew in the intervention group, 2 did not participate in the health campaign, and the other 2 did not continue their health education. The patient in the control group, on the other hand, left the area after enrollment into the study. The analyzable study patients, however, still comprised 126 patients, including 63 receiving mHealth education and 63 receiving usual care. Among these study patients, the mean (SD) age was 57.97 (15.03) years and 57.32 (14.37) years for the control and intervention groups, respectively. Among the 63 study patients in each group, 71% (n=45) were female in the control group, whereas 60% (n=38) were female in the intervention group; 67% (n=42) were housewives in the control group, whereas 56% (n=35) were housewives in the intervention group; and 79% (n=50) were married in the control group, whereas 71% (n=45) were married in the intervention group. Furthermore, comparisons between the control and intervention groups in 4 other categories show the following differences: being illiterate at 40% (n=25) versus 48% (n=30), income <US $100/month at 14% (n=9) versus 22% (n=14), present tobacco user at 13% (n=8) versus 16% (n=10), and current smokeless tobacco user at 43% (n=27) versus 30% (n=19). These characteristics did not differ significantly between the control and intervention groups. Except for hip circumference, no outcome indicators differed significantly at baseline. Baseline details are given in Multimedia Appendix 3, and patient flow through in the trial is shown in Figure 1.

The analyses included repeated measures such as ANOVA, and the results were observed to be significantly different for within groups, between groups, and the interaction of group × time factor in terms of knowledge score. QOL on average changed more favorably in the intervention group than in the control group, but the difference largely failed to achieve statistical significance. Diastolic BP and BMI showed significant differences arising from within groups and the interaction of group × time factor; food salinity and hip circumferences showed significant differences arising from within groups and between groups. Moreover, Systolic BP and waist circumference showed significant differences from within groups. However, no significant differences were found arising from within groups, between groups, and the interactions of group × time in terms of urine salinity and MUAC (Table 1).

Using the generalized estimating equation, knowledge score was considerably increased; however, food salinity and hip circumference were significantly decreased in the intervention group compared to the control group in both the unadjusted and adjusted models. However, waist circumference was considerably lower in the intervention group than the control group in the adjusted model (Table 2).

Regarding the laboratory findings, from baseline to 6 months, the mean (SD) FBS decreased by 0.51 (3.77) mmol/L in the intervention group and by 0.10 (1.44) mmol/L in the control group (*P*=.03); however, BUN increased by 3.64 (7.17) mg/dL in the intervention group and by 1.68 (10.10) mg/dL in the control group (*P*=.01). No other laboratory parameters showed any significant changes over the 6-month duration of the study (Table 3).

**Table 1:** Changes in the study outcomes between the intervention group and control group over time (2-way repeated measures ANOVA test was performed).

Characteristic, group			Baseline, mean (SD)		At 3 months, mean (SD)		At 6 months, mean (SD)			*P* value							
										Within groups			Between groups			Interaction	
**Knowledge score (%)**											<.001			<.001			<.001
	Control	29.89 (18.81)		45.96 (19.30)		42.06 (17.06)											
	Intervention	27.78 (18.34)		68.98 (14.25)		70.76 (13.12)											
**Quality of life (EQ-5D-5L score)**											.83			.21			.91
	Control	0.75 (0.14)		0.76 (0.14)		0.75 (0.14)											
	Intervention	0.78 (0.14)		0.78 (0.13)		0.78 (0.12)											
**Systolic blood pressure (mmHg)**											<.001			.18			.05
	Control	143.73 (24.22)		139.10 (21.89)		131.25 (19.05)											
	Intervention	143.43 (24.12)		130.96 (18.21)		126.55 (18.18)											
**Diastolic blood pressure (mmHg)**											<.001			.79			.001
	Control	88.37 (12.80)		88.20 (12.88)		82.19 (11.27)											
	Intervention	91.38 (12.93)		84.55 (10.70)		81.31 (11.99)											
**BMI (kg/m^2^)**											.01			.13			.02
	Control	25.13 (3.49)		24.71 (3.60)		24.86 (3.73)											
	Intervention	23.83 (4.43)		23.84 (4.40)		23.77 (4.45)											
**Urine salinity (g)**											.96			.57			.40
	Control	10.27 (2.79)		10.20 (2.73)		10.71 (3.24)											
	Intervention	10.64 (3.26)		10.80 (2.97)		10.40 (3.65)											
**Food salinity (g)**											.001			.001			.19
	Control	0.65 (0.23)		0.69 (0.30)		0.62 (0.18)											
	Intervention	0.62 (0.20)		0.61 (0.22)		0.49 (0.15)											
**Mid-upper arm circumference (cm)**											.11			.17			.21
	Control	28.11 (3.17)		27.89 (3.20)		28.00 (3.21)											
	Intervention	27.36 (3.39)		27.30 (3.40)		27.03 (3.16)											
**Waist circumference (cm)**											.003			.15			.51
	Control	87.12 (11.03)		86.88 (10.95)		86.89 (11.01)											
	Intervention	84.18 (11.37)		84.08 (11.35)		84.01 (11.32)											
**Hip circumference (cm)**											.03			.02			.34
	Control	93.72 (6.96)		93.55 (6.88)		93.57 (6.91)											
	Intervention	90.61 (8.75)		90.53 (8.71)		90.29 (8.89)											

**Table 2:** Changes in the study outcomes between the intervention group and control group over time (using generalized estimating equation).

Characteristic	Unadjusted coefficient (95% CI)	*P* value	Adjusted^a^ Coefficient (95% CI)	*P* value
Knowledge score (%)	16.53 (12.06-21.00)	<.001	15.95 (11.76-20.14)	<.001
Quality of life (EQ-5D-5L score)	0.02 (–0.01 to 0.07)	.20	0.019 (–0.02 to 0.05)	.36
Systolic blood pressure (mmHg)	–4.37 (–10.71 to 1.95)	.17	–4.14 (–10.43 to 2.14)	.19
Diastolic blood pressure (mmHg)	–0.5 (–4.19 to 3.170)	.78	–1.05 (–4.67 to 2.56)	.56
BMI (kg/m^2^)	–1.08 (–2.47 to 0.30)	.12	–1.01 (–2.26 to 0.23)	.11
Urine salinity (g)	0.22 (–0.53 to 0.97)	.56	0.15 (–0.61 to 0.92)	.69
Food salinity (g)	–0.08 (–0.12 to –0.03)	.001	–0.08 (–0.12 to –0.03)	.001
MUAC^b^ (cm)	–0.77 (–1.87 to 0.32)	.16	–0.86 (–1.86 to 0.14)	.09
Waist circumference (cm)	–2.87 (–6.74 to 0.99)	.14	–3.83 (–7.21 to –0.46)	.02
Hip circumference (cm)	–3.13 (–5.86 to –0.40)	.02	–3.09 (–5.60 to –0.58)	.01

^a^Adjusted in a generalized estimating equation model for age, gender, education, marital status, and occupation.

^b^MUAC: mid-upper arm circumference.

**Table 3:** Changes of the laboratory values, from baseline to 6 months, between the intervention group and control group (intention-to-treat analysis was performed).

Variable	Intervention (N=63)			Control (N=63)			*P* value
	Mean (SD)	95% CI	Mean (SD)		95% CI		
FBS^a^ (mmol/L)	–0.51 (3.77)	–1.46 to 0.44	–0.10 (1.44)		–0.46 to 0.26	.03^b^	
Serum cholesterol (mg/dL)	–14.22 (27.58)	–21.17 to –7.27	–9.76 (24.57)		–15.95 to –3.57	.34^c^	
Serum creatinine (mg/dL)	0.11 (0.18)	0.07-0.16	0.11 (0.21)		0.06-0.17	.63^b^	
eGFR^d^ (mL/min/1.73 m^2^)	–6.62 (9.81)	–9.09 to –4.15	–6.21 (8.95)		–8.46 to –3.95	.80^c^	
Serum HDL-c^e^ (mg/dL)	–1.36 (8.94)	–3.62 to 0.89	–2.16 (9.12)		–4.46 to 0.14	.58^b^	
Serum triglyceride (mg/dL)	–18.82 (149.36)	–56.44 to 18.79	2.71 (83.51)		–18.32 to 23.75	.75^b^	
Serum albumin (g/dL)	0.07 (0.18)	0.02-0.12	0.07 (0.17)		0.03-0.11	.89^b^	
Hemoglobin (g/dL)	–0.37 (0.82)	–0.58 to –0.16	–0.52 (0.67)		–0.69 to –0.35	.65^b^	
BUN^f^ (mg/dL)	3.64 (7.17)	1.84-5.45	1.68 (10.10)		–0.86 to 4.22	.01^b^	
Serum uric acid (mg/dL)	0.30 (0.94)	0.06-0.53	0.19 (1.01)		–0.06 to 0.45	.35^b^	
ACR^g^ (mg/g)	–86.80 (462.59)	–203.30 to 29.70	–78.08 (212.29)		–131.55 to –24.62	.27^b^	

^a^FBS: fasting blood sugar.

^b^Mann-Whitney *U* test.

^c^Independent 2-tailed *t* test.

^d^eGFR: estimated glomerular filtration rate.

^e^HDL-c: high-density lipoprotein cholesterol.

^f^BUN: blood urea nitrogen.

^g^ACR: albumin-to-creatinine ratio.

## Discussion

### Principal Findings

To our knowledge, this study is the first trial assessing the outcomes of a health education intervention using a nephrologist-facilitated health campaign and CHW-conducted health education using mHealth technology on patients with CKD in Bangladesh. In this single-center, randomized trial of patients with CKD in stages 1-3, health education through a nephrologist-facilitated health campaign and CHW-conducted mHealth education improved the patient knowledge status when compared with usual patient care. In addition, mHealth can significantly increase disease knowledge in patients with CKD. The effectiveness of CKD campaigns in raising CKD awareness and boosting motivation for healthy lifestyle changes to reduce CKD-related complications seems promising. Patients can rely on nephrologists as they are a trusted channel for delivering health education on CKD and related risk factors. Most LMIC have a scarcity of nephrologists, particularly in rural areas; therefore, policy makers should prioritize this leading health problem while formulating appropriate intervention strategies. On the other hand, CHWs play an important role in delivering health education through mHealth [30], because they have the ability to develop interventions and sustain community well-being [31], especially in areas where there are a prevailing shortage of registered physicians and nurses.

It is crucial for patients with CKD to keep their BP under control as hypertension is a major risk factor for the development and progression of CKD. In our analysis, the intervention group had decreased systolic and diastolic BP at the end of the study. Weekly BP measurements, in addition to health education, might also have been influential for this study’s patients. Studies have documented that regular BP monitoring reduces systolic and diastolic BP significantly when compared to usual care [32]. Our findings are comparable with other similar studies that have been undertaken in a range of settings [13,32]. An RCT in rural India showed that a 3-month health education intervention reduced BP in the intervention group compared to the control group [13]. According to a research study, one-third of Bangladeshis have never monitored their BP and have no idea how to control it [14]. Weekly BP checks may alert patients to their BP status, motivating them to better control their diastolic BP. It has also been hypothesized that if patients are aware of their weekly BP levels and know the risk of hypertension with CKD, then they may be more likely to comply with medical therapy in the longer term. In the intervention group, urine salinity remained unchanged despite considerable reductions in BP and dietary salinity. To decrease salt intake, people must regulate their daily lives once they have gained knowledge, which necessitates ongoing community education as well as changes in food business policies.

Patients with diabetes and CKD are at a higher risk of cardiovascular disease and renal failure. Comprehensive education is essential for empowering patients with diabetes and CKD to self-manage their health status [33]. Our effective health education improved patients’ knowledge and awareness about bringing changes in lifestyle and maintaining healthy dietary practices in particular. We found that FBS level was significantly reduced among the intervention group at the end of the 6-month intervention period. According to a study conducted among patients with diabetes in rural China [34], health education enhanced diabetes knowledge and significantly reduced FBS in the intervention group compared to the control group. Related to the BMI, waist and hip circumferences also showed a decreasing trend at the end of 6 months.

The intervention group’s BUN level increased significantly when compared to the control group, which is a notable finding in our study. In patients with CKD, BUN is a marker of the retention of nitrogenous uremic solutes, which are predominantly obtained from protein consumption [35]. Increased protein consumption is the most common extrarenal cause of BUN elevation for patients in the early stage of CKD. Nutritional health education is strongly recommended to achieve a positive outcome.

This study observed a favorable trend of QOL improvement in the intervention group compared to the control group; however, no significant improvement was observed. Health education has a strong favorable influence on all health-related QOL metrics in patients undergoing hemodialysis. However, in our analysis, patients with CKD in stages 1-3 were included, and the 6-month trial duration could be the primary explanation for these poor outcomes. Overall, our data are in line with the concept that health education may improve QOL, even though our study patients in the intervention group were illiterate. These, once again, could be linked to poor urine salinity and MUAC outcomes. Improving QOL, urine salinity, and MUAC in this population may be a challenging goal, and perhaps, multiple interventions with longer duration would be the best approach to the problem.

### Limitations and Strengths

This study’s limitations include a small sample size and a 6-month intervention and follow-up period, in which a longer follow-up period could generate more accurate results. Our study patients were selected randomly from the 3 unions of the Mirzapur subdistrict, and this does not represent the entire rural and peri-urban population with CKD. Moreover, data contamination by neighbors and family members was very plausible; however, the CHWs obtained a verbal agreement from the study patients not to share any study details with their neighbors or other family members. Furthermore, since the participants provided their own data, the outcome assessment was not blinded. The strengths of the study include the facilitation of the health campaign by a nephrologist, and the CHWs’ provision of health education through mobile phone calls in the patients’ native language (Bengali). Health education materials were developed using the same native language for better understanding even with the patients’ minimum technical knowledge and skills. Furthermore, the study’s strengths are the unbiased systematic sampling approach used to recruit patients and the standard laboratory facility used to identify patients with CKD.

### Practical Implications

Integrating nephrologists and CHWs for health education may enhance the knowledge, glycemic control, and hypertension care of patients with CKD in rural and peri-urban Bangladesh. A CKD campaign and the use of mHealth technology would be substantial advantages for the target populations to minimize CKD risk factors. BP monitoring should be included in the routine care of patients with hypertensive CKD to control their BP.

### Conclusions

The health education intervention through a campaign and mHealth technologies demonstrated the potential for improving CKD knowledge among patients with CKD. Education campaigns may have potential for improving FBS and BP among patients with CKD. Both the positive outcomes of health education and weekly BP monitoring interventions on patients with CKD provide evidence for the potential scaling up of these interventions in Bangladesh and possibly other LMIC, especially in rural and peri-urban settings.

## Appendix

Multimedia Appendix 1 Study activities.

Multimedia Appendix 2 Contents of the mobile health education (over a mobile phone call lasting approximately 10 minutes).

Multimedia Appendix 3 Demographic characteristics and outcome variables among the study participants at baseline.

Multimedia Appendix 4 CONSORT-eHEALTH checklist (V 1.6.2).

## Abbreviations

BP: blood pressure
BUN: blood urea nitrogen
CHW: community health worker
CKD: chronic kidney disease
CONSORT: Consolidated Standards of Reporting Trials
DSS: demographic surveillance system
FBS: fasting blood sugar
LMIC: low- and middle-income countries
mHealth: mobile health
MUAC: mid-upper arm circumference
QOL: quality of life
RCT: randomized controlled trial
SPIRIT: Standard Protocol Items: Recommendations for Interventional Trials
