# Supplementary material for: Chronic Kidney Disease Awareness Campaign and Mobile Health Education to Improve Knowledge, Quality of Life, and Motivation for a Healthy Lifestyle Among Patients With Chronic Kidney Disease in Bangladesh: Randomized Controlled Trial
Source: J Med Internet Res. 2022 Aug 11;24(8):e37314. doi: 10.2196/37314 (PMC9412733; doi:10.2196/37314)
Supplement: Multimedia Appendix 1 [file jmir_v24i8e37314_app1.docx]

**Table S1.** Study activities.

| Time schedule | Intervention group | Control group |
| --- | --- | --- |
| Baseline (first week): Interview + administer questionnaires + laboratory test (blood & urine) | | |
| During first two weeks | 1. CKD health campaign (3 hours’ lecture & discussion by a nephrologist)  2. Provide health education materials (leaflet, short textbook, recording notebook and a 5-gram salt measuring spoon) during campaign | Usual care |
| After 2 weeks to 3rd month times | 1. Mobile education: over a phone call- once a 2-week (5 times)  2. Blood pressure check: once a week | Usual care |
| Intermediate (first week of 4^th^ month): Interview & administer questionnaires | | |
| 4^th^ month to 6^th^ month | 1. Mobile education: over a phone call- once a 2-week (5 times)  2. Blood pressure check: once a week | Usual care |
| Final (at the end of 6^th^ month): Interview + administer questionnaires + laboratory test (blood & urine) | | |
