# Supplementary material for: Chronic Kidney Disease Awareness Campaign and Mobile Health Education to Improve Knowledge, Quality of Life, and Motivation for a Healthy Lifestyle Among Patients With Chronic Kidney Disease in Bangladesh: Randomized Controlled Trial
Source: J Med Internet Res. 2022 Aug 11;24(8):e37314. doi: 10.2196/37314 (PMC9412733; doi:10.2196/37314)
Supplement: Multimedia Appendix 2 [file jmir_v24i8e37314_app2.docx]

**Table S2**. Contents of mHealth education (over mobile phone call approximately 10 minutes).

| Topics | Contents of health education |
| --- | --- |
| Kidney | **Kidneys are bean shaped and positioned near the middle of your back on either side of your backbone. Your kidneys are part of the body’s urine system.**  **Functions:** 1. Remove waste products from the body 2. Remove drugs from the body 3. Balance the body's fluids 4. Release hormones that regulate blood pressure 5. Produce an active form of vitamin D that promotes strong and healthy bones 6. Control the production of red blood cells |
| Major risk factors for kidney disease | 1. Diabetes 2. High blood pressure 3. Family history of kidney disease, diabetes or high blood pressure 4. Age 50 years or above 5. Obesity 6. Long time use of painkillers such as aspirin, ibuprofen etc. 7. Chronic kidney infection 8. Kidney stone 9. Smoking etc. |
| Some ways to protect kidneys | 1. Keep blood sugar, blood pressure, and cholesterol under control 2. Lose weight, if needed 3. Eat healthy meals 4. Take all medicines as prescribed 5. Get regular exercise 6. Don’t smoke 7. Avoid some over-the-counter medicines (such as aspirin, or ibuprofen) because they can harm kidneys |
| Diabetes | **Diabetes damages your kidney. Managing blood sugar level slows the kidney damage. Advise:**   1. Take a healthy diet 2. Keep a healthy body weight 3. Exercise at least 30 minutes of moderate physical exercise on 5 days a week 4. Take medicine regularly if prescribed 5. Monitor your blood sugar regularly |
| Hypertension | **Getting your blood pressure back to normal can reduce your kidney damage and some blood pressure tablets actually protect your kidney. Advise:**   1. Reduce salt intake: Excess salt in your body causes your blood pressure to go up. This damages all your blood vessels and increases the risk of heart attack and stroke. Please take salt 5gm/day or less (1 tea spoon) 2. Check blood pressure in regular interval. If possible then buy a blood pressure monitor and measure your blood pressure at home. This can allow you to keep records of your blood pressure and you can see if your blood pressure changes over time 3. Take medicine regularly if prescribed |
