# Supplementary material for: Chronic Kidney Disease Awareness Campaign and Mobile Health Education to Improve Knowledge, Quality of Life, and Motivation for a Healthy Lifestyle Among Patients With Chronic Kidney Disease in Bangladesh: Randomized Controlled Trial
Source: J Med Internet Res. 2022 Aug 11;24(8):e37314. doi: 10.2196/37314 (PMC9412733; doi:10.2196/37314)
Supplement: Multimedia Appendix 3 [file jmir_v24i8e37314_app3.docx]

**Table S3**. Demographic characteristics among the study participants.

| Characteristics | Control group; n=63 (%) | Intervention group; n=63 (%) | *P*-value |
| --- | --- | --- | --- |
| Age in years (mean±SD) | 57.97±15.03 | 57.32±14.37 | .80 |
| Female | 45 (71.4) | 38 (60.3) | .25 |
| Illiterate | 25 (39.7) | 30 (47.6) | .47 |
| Housewife | 42 (66.7) | 35 (55.6) | .28 |
| Married | 50 (79.4) | 45 (71.4) | .48 |
| Income (<USD100/month) | 9 (14.3) | 14 (22.2) | .35 |
| Present tobacco smoker | 8 (12.7) | 10 (15.9) | .79 |
| Present smokeless tobacco user | 27 (42.9) | 19 (30.2) | .19 |
