# Supplementary material for: Chronic Kidney Disease Awareness Campaign and Mobile Health Education to Improve Knowledge, Quality of Life, and Motivation for a Healthy Lifestyle Among Patients With Chronic Kidney Disease in Bangladesh: Randomized Controlled Trial
Source: J Med Internet Res. 2022 Aug 11;24(8):e37314. doi: 10.2196/37314 (PMC9412733; doi:10.2196/37314)
Supplement: Multimedia Appendix 4 [file jmir_v24i8e37314_app4.pdf]

CONSORT-EHEALTH Checklist V1.6.2 Report

Manuscript Number: 37314

(based on CONSORT-EHEALTH V1.6), available at [http://tinyurl.com/consort-ehealth-v1-6].

Date completed

7/16/2022 3:18:47

by

Mohammad Habibur Rahman Sarker

A CKD awareness campaign and mHealth education to improve knowledge, quality of life, and motivation for a healthy lifestyle among CKD patients in Bangladesh: A randomized controlled trial.

TITLE

1a-i) Identify the mode of delivery in the title

A CKD awareness campaign and mHealth education to improve knowledge, quality of life, and motivation for a healthy lifestyle among CKD patients in Bangladesh: A randomized controlled trial.

1a-ii) Non-web-based components or important co-interventions in title

1a-iii) Primary condition or target group in the title

Chronic Kidney Diseases patients

ABSTRACT

1b-i) Key features/functionalities/components of the intervention and comparator in the METHODS section of the ABSTRACT

Adults individual with CKD (stages 1-3) were enrolled in November 2020 and randomly assigned to one of two groups: intervention or control. The intervention group received health education through a CKD awareness campaign and mHealth technologies, whereas the control group received standard treatment, and was observed for 6 months

1b-ii) Level of human involvement in the METHODS section of the ABSTRACT

1b-iii) Open vs. closed, web-based (self-assessment) vs. face-to-face assessments in the METHODS section of the ABSTRACT

1b-iv) RESULTS section in abstract must contain use data

1b-v) CONCLUSIONS/DISCUSSION in abstract for negative trials

INTRODUCTION

2a-i) Problem and the type of system/solution

Yes, we did not face any problem

2a-ii) Scientific background, rationale: What is known about the (type of) system

Yes. CKD education in the early stages could be an integral part of patient management and reduction of the related risk factors, slowing down the progression of the disease, and the need for such education is greater in rural and peri-urban areas

Does your paper address CONSORT subitem 2b?

Yes. the present study aims to evaluate the outcome of a health education intervention designed to enhance knowledge, health-related QOL, and motivation about healthy lifestyle among rural and peri-urban adults suffering from CKD (stage 1-3).

METHODS

3a) CONSORT: Description of trial design (such as parallel, factorial) including allocation ratio

This study was a community-based, single-centered, prospective, open-label, two-arm (1:1), randomized control trial.

3b) CONSORT: Important changes to methods after trial commencement (such as eligibility criteria), with reasons

No, we did not made any changes after trial commencement.

3b-i) Bug fixes, Downtimes, Content Changes

4a) CONSORT: Eligibility criteria for participants

Yes, inclusion and exclusion criteria was used to select the participants.

4a-i) Computer / Internet literacy

4a-ii) Open vs. closed, web-based vs. face-to-face assessments:

Yes (mobile based) health education was provided.

4a-iii) Information giving during recruitment

4b) CONSORT: Settings and locations where the data were collected

Mirjzapur subdistrict, Tangail, Bangladesh

4b-i) Report if outcomes were (self-)assessed through online questionnaires

Health education was provided through mHealth

4b-ii) Report how institutional affiliations are displayed

5) CONSORT: Describe the interventions for each group with sufficient details to allow replication, including how and when they were actually administered

5-i) Mention names, credential, affiliations of the developers, sponsors, and owners

5-ii) Describe the history/development process

5-iii) Revisions and updating

5-iv) Quality assurance methods

5-v) Ensure replicability by publishing the source code, and/or providing screenshots/screen-capture video, and/or providing flowcharts of the algorithms used

5-vi) Digital preservation

5-vii) Access

Not applicable as the participants did not need access to platform or internet.

5-viii) Mode of delivery, features/functionalities/components of the intervention and comparator, and the theoretical framework

Health education for CKD patients

5-ix) Describe use parameters

5-x) Clarify the level of human involvement

5-xi) Report any prompts/reminders used

Yes, we have called all the participants.

5-xii) Describe any co-interventions (incl. training/support)

Yes, training was provided to the community health workers

6a) CONSORT: Completely defined pre-specified primary and secondary outcome measures, including how and when they were assessed

Yes, primary and secondary outcomes were well defined.

6a-i) Online questionnaires: describe if they were validated for online use and apply CHERRIES items to describe how the questionnaires were designed/deployed

6a-ii) Describe whether and how “use” (including intensity of use/dosage) was defined/measured/monitored

6a-iii) Describe whether, how, and when qualitative feedback from participants was obtained

**6b) CONSORT: Any changes to trial outcomes after the trial commenced, with reasons**		
Mirjzapur subdistrict, Tangail, Bangladesh		
**7a) CONSORT: How sample size was determined**		
**7a-i) Describe whether and how expected attrition was taken into account when calculating the sample size**		
**7b) CONSORT: When applicable, explanation of any interim analyses and stopping guidelines**		
Yes, primary and secondary outcomes were well defined.		
**8a) CONSORT: Method used to generate the random allocation sequence**		
Permuted block randomization technique was performed using a block size of six based on a computer-generated random number sequence.		
**8b) CONSORT: Type of randomisation; details of any restriction (such as blocking and block size)**		
Permuted block randomization technique was performed using a block size of six based on a computer-generated random number sequence.		
**9) CONSORT: Mechanism used to implement the random allocation sequence (such as sequentially numbered containers), describing any steps taken to conceal the sequence until interventions were assigned**		
Allocation was concealed in identical sealed envelopes that were only opened when the study patient was ready for enrolment under the supervision of the principal investigator		
**10) CONSORT: Who generated the random allocation sequence, who enrolled participants, and who assigned participants to interventions**		
An experienced statistician, not involved in the study in any way, prepared the randomization table and listed study patients' numbers with corresponding intervention allocations for CKD patients in sequentially numbered sealed envelopes according to the randomization schedule to correspond to the serial number of the CKD patients		
**11a) CONSORT: Blinding - If done, who was blinded after assignment to interventions (for example, participants, care providers, those assessing outcomes) and how**		
**11a-i) Specify who was blinded, and who wasn't**		
This study was a parallel-group (1:1) randomized controlled trial in the Mirzapur sub-district of Bangladesh that compared two groups of CKD patients. Adults individual with CKD (stages 1-3) were enrolled in November 2020 and randomly assigned to one of two groups: intervention or control		
**11a-ii) Discuss e.g., whether participants knew which intervention was the "intervention of interest" and which one was the "comparator"**		
**11b) CONSORT: If relevant, description of the similarity of interventions**		
It was not a placebo control trial.		
**12a) CONSORT: Statistical methods used to compare groups for primary and secondary outcomes**		
For the baseline assessment, continuous variables were compared by t-test or Man-Whitney U test, and categorical variables were compared by the chi-square (X2) test. Multiple comparisons were performed by two-way repeated measure ANOVA test for the evaluation of the outcome variables such as CKD knowledge questionnaire and physical measurements and QOL at baseline, three months, and six months.		
**12a-i) Imputation techniques to deal with attrition / missing values**		
Intention treat analysis were performed.		
**12b) CONSORT: Methods for additional analyses, such as subgroup analyses and adjusted analyses**		
Not applicable as we did not perform any sub group analysis.		
RESULTS		
**13a) CONSORT: For each group, the numbers of participants who were randomly assigned, received intended treatment, and were analysed for the primary outcome**		
Yes, intention to treat analysis was performed		
**13b) CONSORT: For each group, losses and exclusions after randomisation, together with reasons**		
A total enrolled of 126 patients (control group, n=63, intervention group, n=63) were enrolled in the study. Of them, five patients were withdrawn from further participation during the follow-up period. Among them, four were from the intervention group and one from the control group. Two patients in the intervention group did not participate in the health campaign, and the other two did not continue their health education. The only patient in the control group, on the other hand, left the area after enrolment into the study".		
**13b-i) Attrition diagram**		
**14a) CONSORT: Dates defining the periods of recruitment and follow-up**		
The total study duration was one year; during that one year, the intervention duration was six months, starting from mid-November 2020 and completed in May 2021		
**14a-i) Indicate if critical "secular events" fell into the study period**		
**14b) CONSORT: Why the trial ended or was stopped (early)**		
Not applicable as it was not stopped (early).		
**15) CONSORT: A table showing baseline demographic and clinical characteristics for each group**		
Yes, baseline characteristics was provided.		
**15-i) Report demographics associated with digital divide issues**		
Yes. Age, gender, education was provided in demographics characteristics was provided.		
**16a) CONSORT: For each group, number of participants (denominator) included in each analysis and whether the analysis was by original assigned groups**		
**16-i) Report multiple "denominators" and provide definitions**		
Yes, clearly defined the denominators.		
**16-ii) Primary analysis should be intent-to-treat**		
**17a) CONSORT: For each primary and secondary outcome, results for each group, and the estimated effect size and its precision (such as 95% confidence interval)**		
Yes. Estimated effect size and its precision.		
**17a-i) Presentation of process outcomes such as metrics of use and intensity of use**		
**17b) CONSORT: For binary outcomes, presentation of both absolute and relative effect sizes is recommended**		
For binary outcomes, presentation of both absolute and relative effect sizes was measured		
**18) CONSORT: Results of any other analyses performed, including subgroup analyses and adjusted analyses, distinguishing pre-specified from exploratory**		
Repeated measure ANOVA and Generalized Estimating Equation (GEE) was performed		
**18-i) Subgroup analysis of comparing only users**		
**19) CONSORT: All important harms or unintended effects in each group**		
No any important harm was observed.		
**19-i) Include privacy breaches, technical problems**		
**19-ii) Include qualitative feedback from participants or observations from staff/researchers**		
DISCUSSION		
**20) CONSORT: Trial limitations, addressing sources of potential bias, imprecision, multiplicity of analyses**		
**20-i) Typical limitations in ehealth trials**		
Data contamination by neighbors and family members was very plausible		
**21) CONSORT: Generalisability (external validity, applicability) of the trial findings**		
**21-i) Generalizability to other populations**		
**21-ii) Discuss if there were elements in the RCT that would be different in a routine application setting**		
**22) CONSORT: Interpretation consistent with results, balancing benefits and harms, and considering other relevant evidence**		
**22-i) Restate study questions and summarize the answers suggested by the data, starting with primary outcomes and process outcomes (use)**		
Health education through a nephrologist facilitated health campaign and CHWs conducted mHealth education improved the patient knowledge status when compared with usual patient care		
**22-ii) Highlight unanswered new questions, suggest future research**		

Other information		
**23) CONSORT: Registration number and name of trial registry**		
ClinicalTrials.gov NCT04094831.		
**24) CONSORT: Where the full trial protocol can be accessed, if available**		
JMIR Res Protoc. 2021 Nov 19;10(11):e30191.doi: 10.2196/30191		
**25) CONSORT: Sources of funding and other support (such as supply of drugs), role of funders**		
Japan's Grants-in-Aid for Scientific Research Program		
**X26-i) Comment on ethics committee approval**		
**x26-ii) Outline informed consent procedures**		
**X26-iii) Safety and security procedures**		
**X27-i) State the relation of the study team towards the system being evaluated**		
